# Supplementary figures and images for: Characterization and deorphanization of RYamide signaling in Aedes aegypti: A potential regulator of hindgut-associated physiology
Source: PLoS One. 2026 Feb 23;21(2):e0342341. doi: 10.1371/journal.pone.0342341 (PMC12928595; doi:10.1371/journal.pone.0342341)

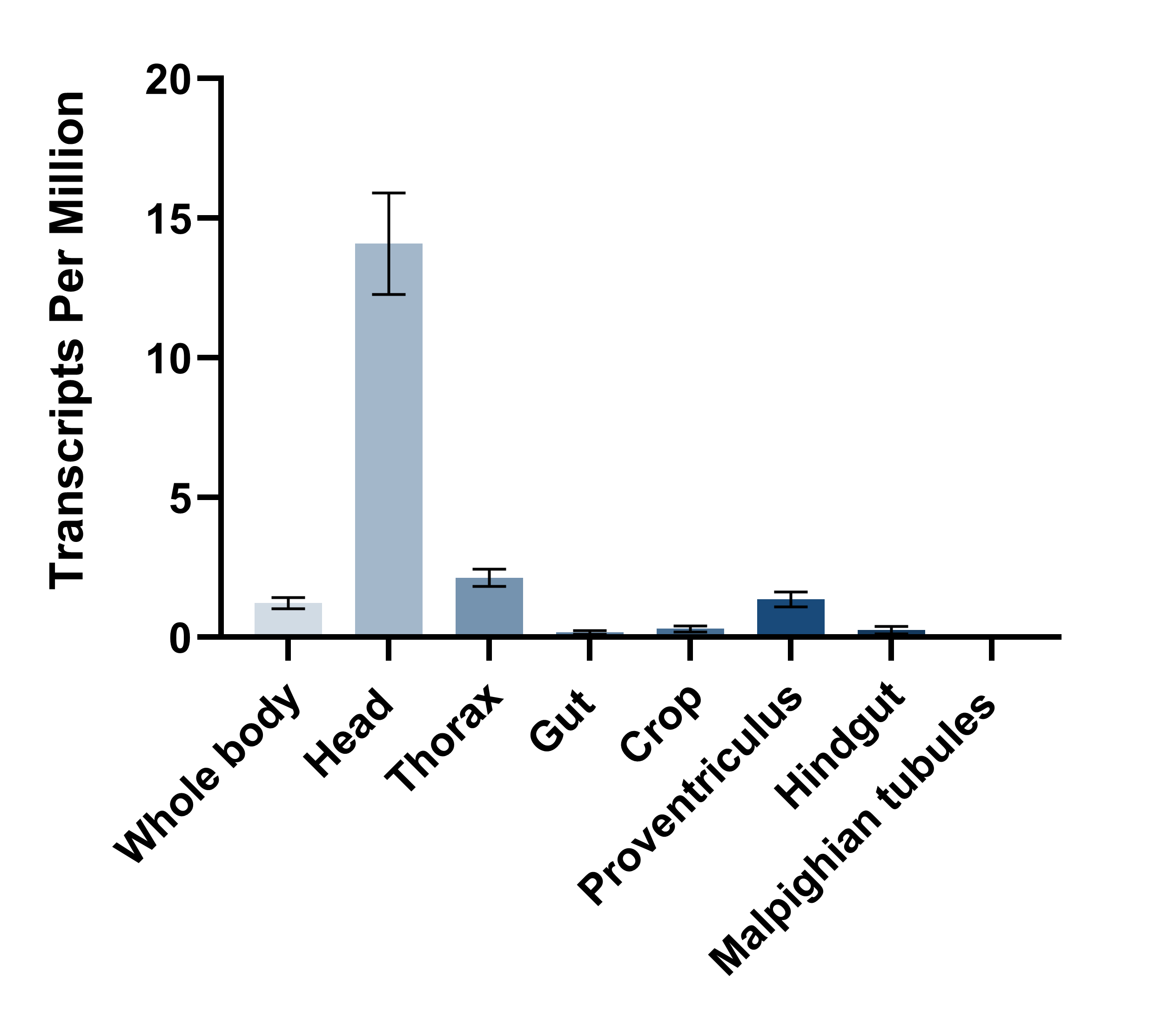

Supplement: S1 Fig — Figure was prepared using available RNA-seq dataset (Hixson et al. 2022). (TIF) [file pone.0342341.s001.tif]

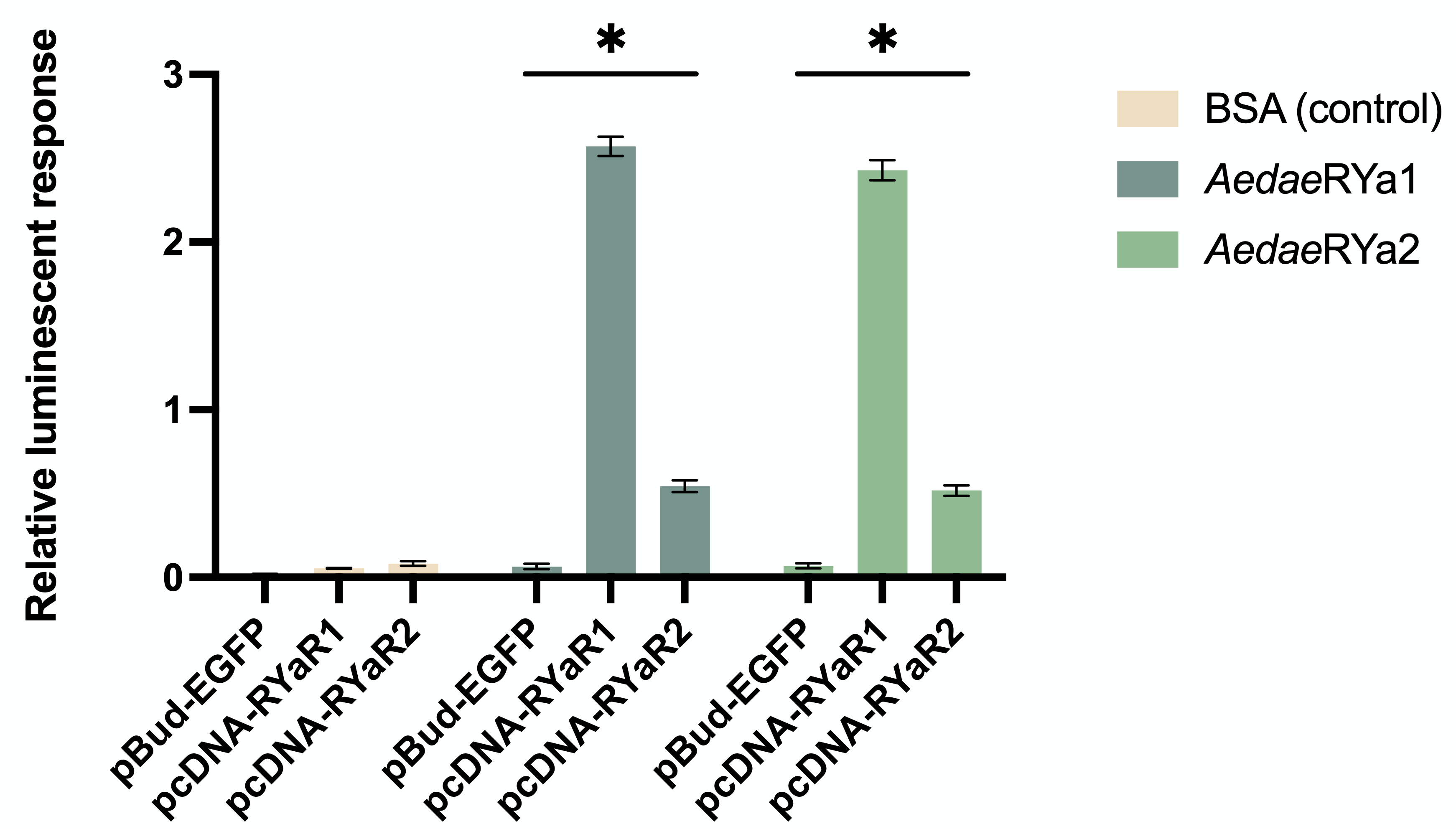

Supplement: S2 Fig — Assay media (BSA) alone and different ligands (10-6M) were applied to cells expressing different expression constructs to validate the receptor activity by comparing the luminescent responses generated via receptor activation. Statistical differences are denoted with asterisk (*), as determined by a two-way ANOVA with Šídák’s multiple comparisons test (p < 0.05). (TIFF) [file pone.0342341.s002.tiff]

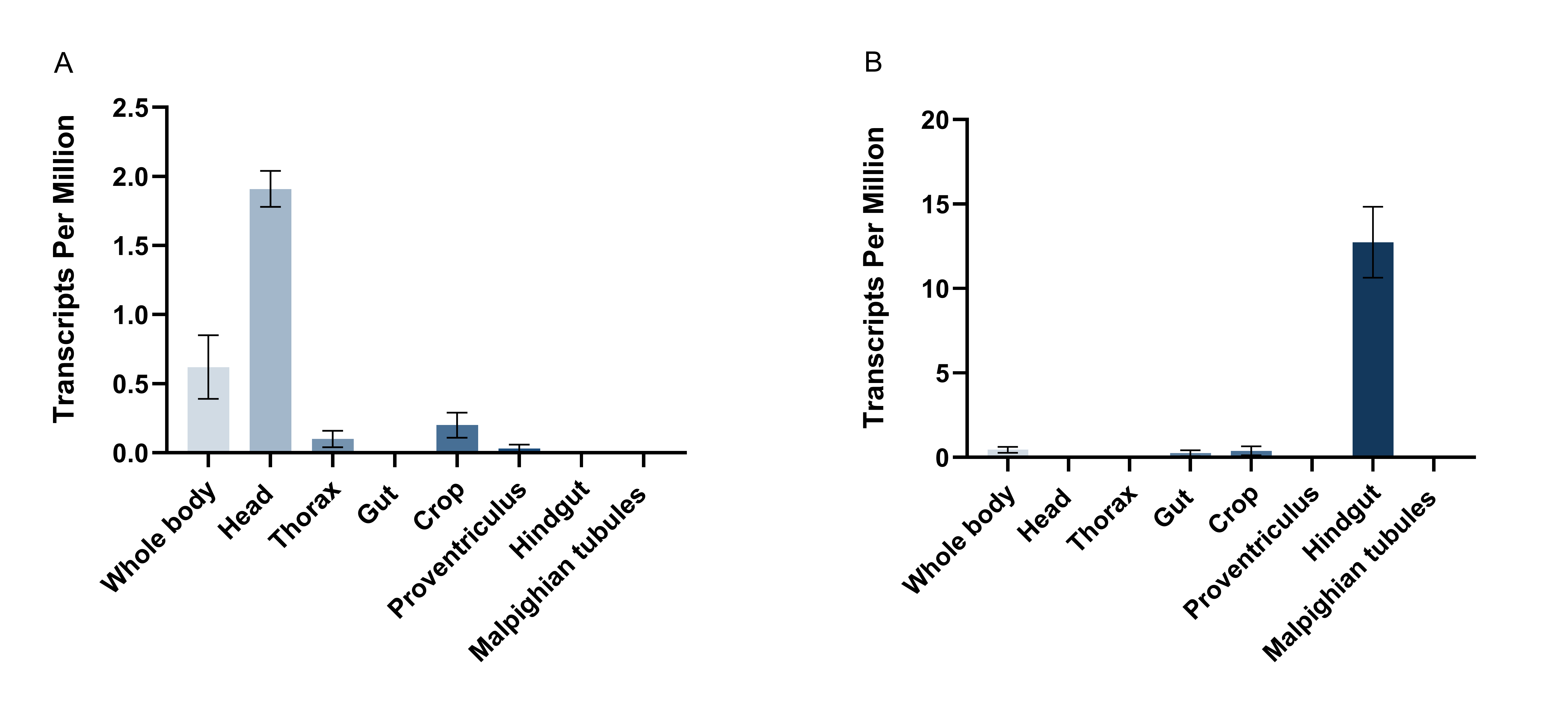

Supplement: S3 Fig — Figure was prepared using available RNA-seq dataset (Hixson et al. 2022). (TIF) [file pone.0342341.s003.tif]

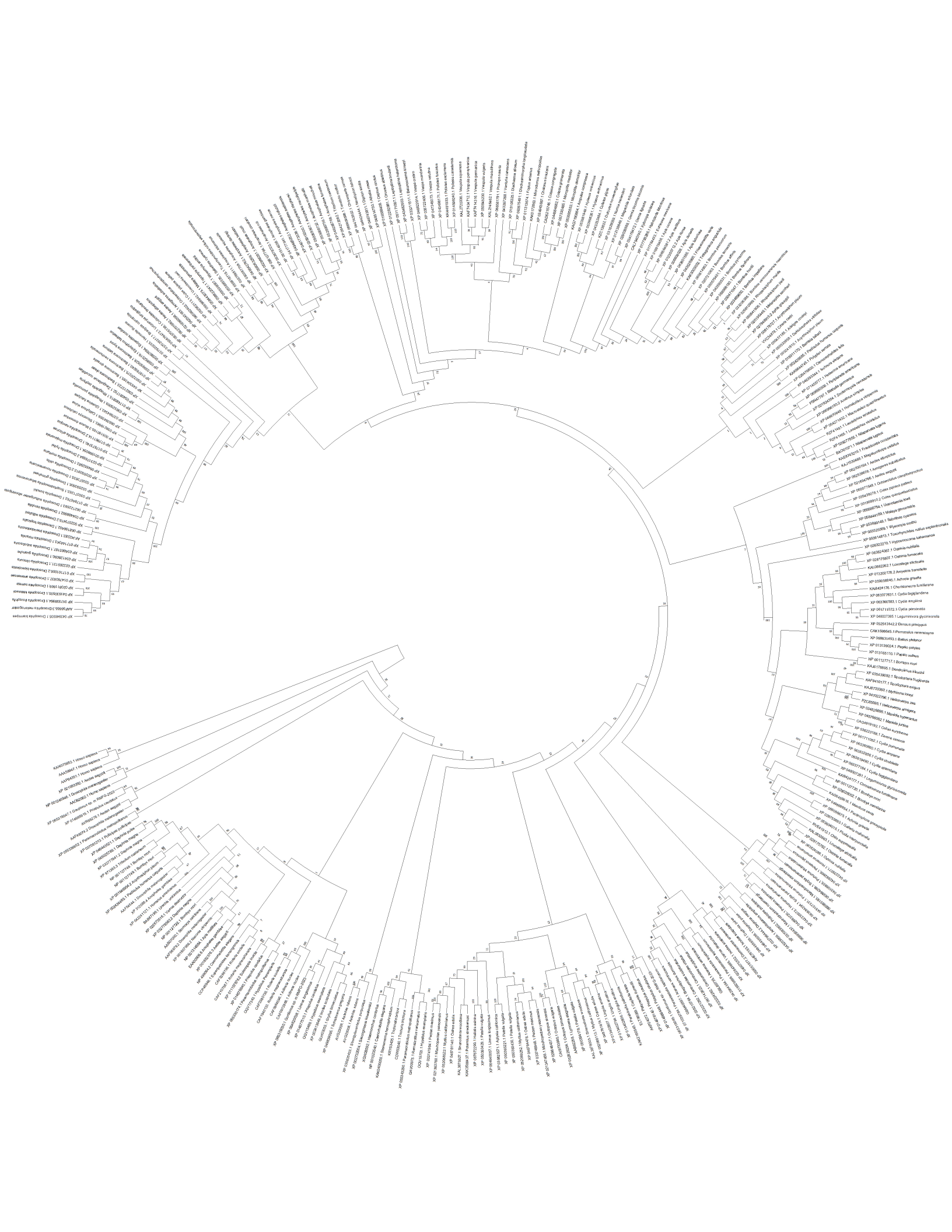

Supplement: S4 Fig — Tree was constructed using maximum-likelihood phylogenetic analysis methods (with 1000 bootstrap replicates). The annotated numbers adjacent to nodes indicate the support percentage for the clustering of related sequences within the respective clade. Tachykinin, NPF and sNPF receptors, as well as NPY receptors of Homo sapiens, were included to demonstrate the evolutionary relationship between vertebrate NPY receptors and invertebrate RYamide, tachykinin, NPF, and sNPF receptors. Homo sapiens NPYR was included in the analysis and imposed as the outgroup. (PDF) [file pone.0342341.s004.pdf]
