# Supplementary material for: Characterization and deorphanization of RYamide signaling in Aedes aegypti: A potential regulator of hindgut-associated physiology
Source: PLoS One. 2026 Feb 23;21(2):e0342341. doi: 10.1371/journal.pone.0342341 (PMC12928595; doi:10.1371/journal.pone.0342341)
Supplement: S1 Table — Geneious Pro Bioinformatics Software was used to design primers. (DOCX) [file pone.0342341.s005.docx]

| Primers | Sequences | Functions |
| --- | --- | --- |
| AAEL017005-Kozak | TTCTGCCGCCACCATGAGCG | ORF cloning of RYaR1 (AAEL017005) for functional receptor assay |
| AAEL017005-Stop | GGTTGTTGTATCACCGTAGC |  |
| AAEL019786-Kozak | AAGCTTGCCACCATGAACTTCACTGCCGAG | ORF cloning of RYaR2 (AAEL019786) for functional receptor assay |
| AAEL019786-Stop | ATTCTAGAATTCAACCCCCTACAACC |  |
| RYa-qPCR-F | CTAATCCTTCTAGTCAGTGCGG | RT-qPCR amplification of *Aedae*RYamide (RYa) |
| RYa-qPCR-R | AGCGGGATCCAAGAAAGAAGC |  |
| RYa_RNAi-F | TGCGCCACCACACTCCATAT | dsRNA synthesis for RYa knockdown |
| RYa_RNAi-R | GGAGATCTTTCCCGCCGTAGA |  |
