# Supplementary material for: Characterization and deorphanization of RYamide signaling in Aedes aegypti: A potential regulator of hindgut-associated physiology
Source: PLoS One. 2026 Feb 23;21(2):e0342341. doi: 10.1371/journal.pone.0342341 (PMC12928595; doi:10.1371/journal.pone.0342341)
Supplement: S2 Table — Highlighted sequences indicate the conservation of residues at the C-terminus. (DOCX) [file pone.0342341.s006.docx]

| **Name** | **Amino acid sequence** |
| --- | --- |
| NPF | SFTDARPQDDPTSVAEAIRLLQELETKHAQHARPRFa |
| 2sNPF-1 | KAVRSPSLRLRFa |
| sNPF-1 | SPSLRLRFa |
| sNPF-2 | APQLRLRFa |
| sNPF-3 | APSQRLRWa |
| FMRFa-1 | SALDKNFMRFa |
| FMRFa-2 | ASKQANLMRFa |
| FMRFa-3 | AGQGFMRFa |
| FMRFa-4 | DSPKNLMRFa |
| FMRFa-8 | GSGNLMRFa |
| FMRFa-9 | AKGNLMRFa |
| FMRFa-11 | MDNNFMRFa |
| Head peptide 1 | (pE)-RP-(hP)-SLKTRFa |
| Myosuppressin | TDVDHVFLRFa |
| Pyrokinin1 (PK1) | AGNSGANSGMWFGPRLa |
| CAPA | pQGLVPFPRVa |
| RYamide 1 | PFFVGSRYa |
| RYamide 2 | NDRFFLGSRYa |

*Note: pE is pyro-glutamic acid; hP is 4-hydroxyproline*
